# Renewed assessment of the risk of emergent advanced cell therapies to transmit neuroproteinopathies

**DOI:** 10.1007/s00401-018-1941-9

**Published:** 2018-11-27

**Authors:** Paul A. De Sousa, Diane Ritchie, Alison Green, Siddharthan Chandran, Richard Knight, Mark W. Head

**Affiliations:** 1 Centre for Clinical Brain Sciences, University of Edinburgh, Chancellors Building, 49 Little France Crescent, Edinburgh, EH16 4SB UK; 2 National CJD Research & Surveillance Unit, Western General Hospital, Crewe Road, Edinburgh, EH4 2XU UK

**Keywords:** Prion, Proteinopathy, Advanced cell therapy, Neurodegenerative disease

## Abstract

The inadvertent transmission of long incubating, untreatable and fatal neurodegenerative prionopathies, notably iatrogenic Creutzfeldt–Jakob disease, following transplantation of cadaver-derived corneas, pituitary growth, hormones and dura mater, constitutes a historical precedent which has underpinned the application of precautionary principles to modern day advanced cell therapies. To date these have been reflected by geographic or medical history risk-based deferral of tissue donors. Emergent understanding of other prion-like proteinopathies, their potential independence from prions as a transmissible agent and the variable capability of scalably manufacturable stem cells and derivatives to take up and clear or to propagate prions, substantiate further commitment to qualifying neurodegenerative proteinopathy transmission risks. This is especially so for those involving direct or facilitated access to a recipient’s brain or connected visual or nervous system such as for the treatment of stroke, retinal and adult onset neurodegenerative diseases, treatments for which have already commenced. In this review, we assess the prospective global dissemination of advanced cell therapies founded on transplantation or exposure to allogeneic human cells, recap lessons learned from the historical precedents of CJD transmission and review recent advances and current limits in understanding of prion and other neurodegenerative disease prion-like susceptibility and transmission. From these we propose grounds for a reassessment of the risks of emergent advanced cell therapies to transmit neuroproteinopathies and suggestions to ACT developers and regulators for risk mitigation and extension of criteria for deferrals.

**Electronic supplementary material:**

The online version of this article (10.1007/s00401-018-1941-9) contains supplementary material, which is available to authorized users.

## Introduction

Over the past 20 years, scientific and medical research communities have made significant preclinical and clinical progress in the development of new “advanced cell therapy “ (ACT) paradigms aspiring to replace, reconstitute, or regenerate diseased or injured tissues, or modulate host-immune responses. Contrary to canonical allogeneic or autologous transplantation of primary or minimally processed tissues such as bone marrow and corneas or pituitary extracts as a source of growth hormone, these new paradigms have entailed more extensive processing of renewable cell sources. This has augmented scope for dissemination and thus potential risk of harm to recipients and the general population. The provenance of cell source material for these encompasses the spectrum of cell developmental ontogeny from early pre-implantation embryos, to fetal, neonatal, and extra-embryonic and adult organ-specific tissues (i.e., pancreas, bone, fat, blood). Each places different constraints and requirements on the design and attributes of the intended ACT. These include the prospective quantity, purity, proliferative capacity, lineage potency, and cellularity of source material and derivative therapeutic products; the necessity for co-administration with bio-compatible materials or augmentation of function by genetic manipulation; the extent to which mechanism of action is understood or defined; and the methods, reagents and assays used in production or quality control.

Concern for ACT biosafety has centered on qualifying the risk of adverse events arising from (1) uncontrolled or unexpected cell growth, behavior or neoplastic transformation, normally qualified through transplantation into animal models; and (2) adventitious pathogen transmission. The greatest concern for the latter is normally diseases for which there are no validated tests available to identify pathogen presence in the absence of visible symptoms of disease, or technology to remove infectivity if detected. Prion diseases of human or animal origin such as genetic, iatrogenic, sporadic or variant Creutzfeldt–Jacobs disease (g/i/s/vCJD), bovine spongiform encephalopathy (BSE), ovine scrapie and chronic wasting disease (CWD) in deer, which result in long incubating, invariably fatal and untreatable neurodegenerative prionopathies, have epitomized concern for such pathogens. Safeguards against these have been addressed on the basis of risk-based donor deferral founded on medical history and geographical likelihood of exposure. Unfortunately, this approach is increasingly challenged by the expanding recognition and global prevalence of prion diseases of one form or another and the increasing mobility of populations. In principle the efficacy of risk-based deferrals is determined by accuracy and sufficiency of information on which donor selection criteria is based. Rising awareness and improved understanding of prion susceptibility and propagation in ex vivo cultivated stem and derivative cells, and latterly evidence of prion-like transmission observed with other neurodegenerative disease-associated proteinopathies, now substantiate a reassessment of the risks that emergent ACT pose to transmit neuroproteinopathies to both recipients and the general population through secondary transmission. The latter is facilitated by the ageing and longer living demographic which recipients represent. In this review, we begin by summarising the current state of allogeneic ACT market authorisations and estimating their global dissemination to date via clinical trial evaluations. For the latter we assess prospective patient participation in trials of therapeutic products derived from distinctive sources differing in source material growth, lineage potency and properties impacting on the design of their use and target indication. We then recap the historical precedents of CJD transmission and review recent advances in understanding of prion and other neurodegenerative disease prion-like susceptibility and transmission. Collectively, we propose these substantiate grounds for renewed assessment of risks of transmitting neurodegenerative disease causing proteinopathies (i.e., neuroproteinopathies). We suggest measures to mitigate such risks through research, product testing and extension of criteria for deferrals.

## Current allogeneic ACT

In principle ACT founded on a strategy of one allogeneic cell source for many recipients carries a greater risk for adventitious pathogen transmission to recipients and secondary infection to the general population, than one to one or autologous transplants. The extent of this may vary in accordance with: (1) how greatly cell source material can be renewed and expanded for dissemination; (2) the nature of the contaminant and the point in the process at which it occurs. The risk of transmitting a neuroproteinopathy will be greater where products are transplanted directly into the brain or central nervous system (CNS) or may have facilitated access to these by virtue of the site of their transplantation (e.g., innervated proximal organs such as the eye) vs transplantation to a peripheral organ. However, injury or inflammation compromising the blood–brain barrier at the time of a transplant or subsequently could in principle elevate the risk of transmission. Further, risks of secondary transmission to the general population, such as via recipient donated blood would either be equal or greater following a peripheral transplant owing to facilitated exposure to the recipient’s circulatory system depending first on the transmission potential of the proteinopathy and infected tissue. The likelihood of transmission to the general population would of course also depend on the eligibility of the recipient to make a tissue donation post-treatment, whether owing to health or precautionary deferral.

Allogeneic ACTs currently under evaluation in clinical studies can be conceptually distinguished on the basis of cell growth and lineage potency, in order of greatest to least: human embryo or induced pluripotent stem cells (hESC/hiPSC), multipotent mesenchymal stromal/stem cells (hMSC) or lineage committed progenitors and differentiated lineage or immortalized cell lines [11, 56, 66, 80, 82]. The latter includes diverse allogeneic cancer cell immunotherapies and vaccines such as major Multi-Histocompatibility Complex antigen matched natural killer or cytolytic T-cells, or inactivated tumor cell vaccines [71]. To date the only allogeneic cell therapy to be granted regulatory authority marketing authorization anywhere in the world is an hMSC treatment for graft versus host disease in New Zealand, Canada and Japan [28].

**Fig. 1:**
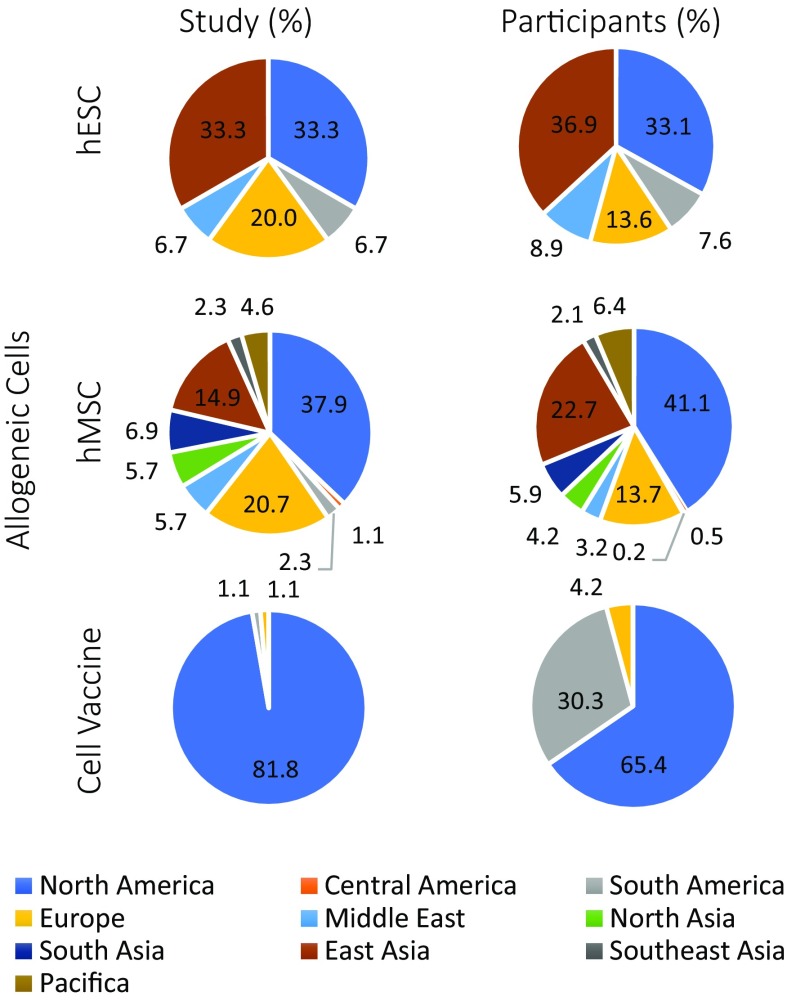
Snapshot of current global clinical evaluation of human allogeneic embryonic, and mesenchymal stem cell and cell vaccine therapies. Inquiry of the international clinical trials registry [13] for recruiting or completed phase 1–4 trials for terms: “Embryo Stem Cells”, “Allogeneic Mesenchymal” or “Allogeneic Cell Line” returned 15, 87 and 11 trials verified to match the search terms, and an estimated participation of 236, 4088 and 356 patients, respectively. Results are depicted as study and participant percentages per worldwide region, with regional definitions defined by clinical.trials.gov mapping algorithm. The specific countries in which trials are/have been conducted are as noted in parenthesis as follows: North America (USA and Canada); Central America (Panama); South America (Brazil, Argentina); Europe (Belgium, Denmark, France, Ireland, Italy, Netherlands, Spain, Sweden, United Kingdom); Middle East (Islamic Republic of Iran, Jordan, Israel); North Asia (Russian Federation, Ukraine, Kazakhstan); South Asia (India, Pakistan); East Asia (China, Republic of Korea, Taiwan); Southeast Asia (Malaysia, Vietnam); Pacifica (Australia). Further details of studies provided in Table S1

To estimate the current relative preponderance of clinical evaluations of conceptually distinct ACT globally we interrogated the international clinical trials registry [13] for allogeneic embryonic, mesenchymal stem cell, or cancer cell vaccine based clinical interventions at phase 1–4 which are recruiting or completed. The outcome of this assessment is summarized in Fig. 1, with links to outcomes of searches in Table S1. Table 1 provides a non-exhaustive exemplar of the spectrum of allogeneic ACT in preclinical development and clinical evaluation. For allogeneic mesenchymal stem cell-based therapies, there are currently 87 trials with an estimated proposed participation of 4088 patients around the world, the greatest in North America (predominantly USA), Europe, and East Asia (predominantly China). These concern treatment of acute tissue injury, chronic degenerative disorders and inflammatory disease based on bone marrow, adipose and extraembryonic tissue sourced cells. The prevalence of hESC and allogeneic cancer cell vaccines is an order of magnitude less. The latter, consisting of 11 studies and proposed cumulative participation of 356 patients, are predominantly in the USA. For hESC 15 trials proposing a total participation of 236 patients have a global distribution akin to hMSC. While trials for an hESC treatment of spinal cord injury and diabetes, and hiPSC based therapies for macular degeneration, have been initiated (reviewed in [83]), those hESC based trials which are currently recruiting or completed largely target retinal disease (11 of 15), with two treating neurodegenerative conditions (Parkinson’s and amyotrophic lateral sclerosis) and one ischemic heart disease. The choice of the eye and retinal degenerative disease early during ACT therapeutic validation is logical given the seriousness of disease, the physical accessibility of this part of the CNS, and the ease with which treatment efficacy can be measured. It is not, however, without a potentially greater risk of facilitating prion or prion-like pathogens to the brain.

**Table 1:** Non-exhaustive exemplar spectrum of allogeneic ACT in preclinical development and clinical evaluation

Primary tissue origin of ACT source material	ACT source material type	ACT product(s) derived from source material	Administration site	Medical indication(s)
Preimplantation embryos, adult peripheral blood cells, dermal fibroblasts	Pluripotent stem cells	Ocular pigmented retinal epithelium	Ocular	Retinal diseases [16, 74–76, 83]^a^
		Brain substantia nigra dopaminergic neurons	Cerebral	Parkinson’s disease [31, 79]^a^
		Brain striatal medium spiny neurons and glia	Cerebral	Huntington’s disease [1]
		Oligodendroglia cells	Cerebral/spinal	Spinal cord injury; amyotrophic lateral sclerosis [63, 79]^a^
		Cardiac progenitor cells	Cardiac	Ischemia heart disease [91]^a^
		Pancreatic insulin secreting cells	Hepatic	Diabetes [10]
		Skin keratinocytes	Dermal	Dermal reconstruction, ulceration [32]
		Vascular endothelial cells	Vascular/central or peripheral tissues	Tissue ischemia [89]
		Mesenchymal stem/stromal cells	Vascular/central or peripheral tissues	Graft vs host disease; disease associated acute and chronic tissue specific or systemic inflammation; tissue ischaemia, tissue reconstruction; as a gene therapy bio-vector [23, 27, 30, 53]
Bone marrow, adipose tissue, dental pulp, placenta	Mesenchymal stem/stromal cells	Multipotent mesenchymal stem/stromal cells	Vascular/central or peripheral tissues	Graft vs host disease; disease associated acute and chronic tissue specific or systemic inflammation; tissue ischemia; tissue reconstruction; as a gene therapy bio-vector^b^
Fatal neural tissue	Neuralstem cells	Non- or immortalised neural stem cells and lineage committed progenitors	Cerebral/Spinal	Spinal cord injury; stroke [44, 59]
Adult peripheral blood cell	Cytolytic T cell; natural killer cells	Cytolytic T-cell or natural killer cells exposed to cancer cell antigens	Vascular/central or peripheral tissues	Cancer causing and post haematopoietic stem cell transplant viral infections; lymphoma; leukaemia; myeloma; glial cell blastoma and sarcoma; intestinal and ependymoblastoma tumors^c^
Cancer	Cancer cell line	Genetically modified cancer cell line to vaccinate recipient immune system	Vascular/central or peripheral tissues	Skin, prostate, breast, brain and lung cancer^d^

^a^Target indications under clinical evaluation specified in ClinicalTrials.gov search Table S1, No. 5 and 6: Macular degeneration; Stargardt’s macular dystrophy; macular degenerative disease; dry age-related macular degeneration; exudative age-related macular degeneration; myopic macular degeneration; geographic atrophy; ischemic heart disease; amyotrophic lateral sclerosis; type 1 diabetes; Parkinson’s disease

^b^Target indications under clinical evaluation specified in ClinicalTrials.gov search Table S1, No. 1 and 2: Adult diseases for refractory scleroderma, endothelial dysfunction, rheumatoid arthritis, cystic fibrosis, post-transplant cytopenia, critical limb ischemia, liver cirrhosis, tibial closed diaphyseal fractures, pediatric inflammatory bowel disease, osteoarthritis, diabetic foot ulcers, anthracycline induced cardiomyopathy, heart failure, dystrophic epidermolysis bullosa, hypoplastic left heart syndrome, asthma, non-cystic fibrosis bronchiectasis, progressive interstitial lung disease, type I diabetes, diabetic nephropathy, non-ischemic dilated cardiomyopathy, type 2 diabetes, osteodysplasia, refractory acute graft vs host, acute respiratory distress syndrome, steroid refractory graft vs host, depression, cerebellar ataxia, aging frailty, osteoarthritis, degenerative disc disease/lumbar back pain, idiopathic Parkinson’s, acute-on-chronic liver failure, Type I diabetes with diabetic ketoacidosis, Werdnig Hoffman Disease (infantile spinal muscular atrophy, type I), acute ischemic stroke, knee osteoarthritis, severe psoriasis, Alzheimer’s disease, cerebral palsy, refractory perianal Crohn’s disease, non-union fracture, multiple sclerosis, prevention of graft vs host disease, burn wounds, sepsis, limbus cornea insufficiency, chronic lung allograft dysfunction, leukemia (acute myeloid, acute lymphoblastic, chronic myelocytic, myeloproliferative, myelodysplastic, myeloma, chronic lymphocytic, Hodgkin’s disease, non-Hodgkin’s), bronchopulmonary dysplasia, peripheral artery disease, peripheral vascular disease, aplastic anemia, abdominal aortic aneurysm, hypercholesterolemia, osteogenesis imperfecta, neomyogenesis in dilated cardiomyopathy, second and third degree burns

^c^Target indications under clinical evaluation specified in GlinicalTrials.gov search Table S1 No. 3 and 4: Epstein-Bar, cytomegalo-, adeno-virus infections; viral infection post hematopoietic stem cell transplant; post-transplant lymphoproliferative disorder; anaplastic astrocytoma, ependymoma meningioma, oligodendroglioma; brain stem glioma; ependymoblastoma; giant cell glioblastoma; glioblastoma; gliovascular; grade III meningioma; meningioma; meningeal hemangiopericytoma; mixed glioma; pineal gland astrocytoma; brain tumor; acute and chronic lymphocytic leukemia; follicular, mantle, B-cell prolymphocytic and diffuse large cell lymphoma; acute myeloid, precursor T-cell lymphoblastic, T-cell prolymphocytic, T-cell large granular lymphocytic leukemia; peripheral t-cell, angioimmunoblastic T-cell, extra nodal NK/T-cell, enteropathy-type intestinal T-cell and hepatosplenic T-cell lymphoma; metastatic solid tumor

^d^Target indications under clinical evaluation specified in ClinicalTrials.gov search Table S1, No. 3 and 4. Melanoma; prostate cancer; breast cancer; neuroblastoma; lung and bronchogenic cancer

## Iatrogenic CJD: a cautionary tale

The transmissibility of CJD presents a series of public health risks, including the risk of secondary transmission through medical or surgical intervention resulting in iatrogenic CJD (iCJD). Historically routes of transmission have included corneal transplantation and neurosurgical instruments, but the largest numbers of cases of iCJD worldwide have been through the use of cadaveric pituitary-derived hormones and human growth hormone-associated CJD (hGH-iCJD) in France, the UK and the USA and dura mater grafting (largely, but not exclusively in Japan) (reviewed by Ref. [8] and summarized in Table 2). Despite the practice of extracting growth hormone from cadaveric pituitaries having ended in the UK in 1985, cases of hGH-iCJD still occur, indicative of the 40 years or more incubation periods possible in these diseases and the disastrous long-term consequences of inadvertent CJD transmission [72]. To this can be added the finding that vCJD (the human form of BSE in cattle) infectivity can be transmitted person-to-person via red cell transfusion (recent update from Ref. [86]) and factor VIII treatment [65].

**Table 2:** Global incidence of iatrogenic prion and prion-like transmission and disease in relation to medical treatment and indication

Medical treatment (site of administration)	Medical indication	Product or mode of transmission	Known or suspected infectious source	Estimated CJD transmission risk and, or number of known cases (geographical location of affected patients)	Unexpected finding of Aβ accumulation in the brain and, or cerebral blood vessels of indicated patient groups (geographical location of affected patients)
Growth hormone therapy (intramuscular)	Primary and secondary pituitary insufficiency	Hormone batches prepared from pooled human cadaveric pituitary glands	Inclusion of tissue(s) from sporadic CJD case(s)	1–10% of treated groups > 230 cases worldwide (Europe, USA) [8]	26/72 growth hormone-associated CJD cases examined (aggregate numbers from four studies conducted in Europe and the USA) [9, 42, 70] 5/12 growth hormone treated patients without CJD (UK) [70]
Dura mater grafting (CNS)	Repair or replacement of dura during neurosurgery	Batches prepared from pooled dura mater tissue from human cadaveric brain	Inclusion of tissue(s) from sporadic CJD case(s)	< 1% of grafted patients (Japan) > 228 cases world-wide (Japan, Europe, USA) [8]	26/36 dura mater-associated CJD cases examined (aggregate numbers from four studies conducted in Japan, Europe and the USA) [9, 26, 34, 46] Case report of dura mater recipient without CJD (Europe) [38]
Neurosurgery, electro-encephalography (CNS)	Neurological disease, and electrophysiological recording	Re-use of contaminated instruments and depth electrodes	Prior use on a patient with sporadic CJD	Six cases world-wide (UK, France, Switzerland) [8]	Eight patients < 55 years of age, without CJD, with a severe cerebral amyloid angiopathy and a history of childhood neurosurgery (Europe, Japan) [43]
Corneal transplantation (eye)	Replacement of damaged or diseased cornea	Individual donated cadaveric human eyes	Donation from unrecognised sporadic CJD case	One definite (USA) and four suspected cases (Japan, Europe, USA) [84]	Not reported
Blood transfusion (intravenous)	Symptomatic anaemia, acute blood loss	Individual units of donated human packed red blood cells	Unrecognised preclinical variant CJD blood donor	Four infections linked to 67 recipients of implicated components from 18 donors (UK). Three cases of typical clinical variant CJD and one asymptomatic case with evidence of infection in the spleen (UK) [62]	Not reported
Factor VIII treatment (intravenous)	Haemophilia	Batches of fractionated and purified pooled blood donations	Inclusion of preclinical variant CJD donor bloods	One out of 17 at risk asymptomatic haemophiliacs tested positive in spleen (UK) [62]	Not reported

Although CJD is very rare, the consequences of its transmissibility are very serious for patients and medical practitioners. CJD is uniformly fatal with long incubation periods and no currently effective prophylaxis or therapy. This places potentially exposed individuals, such as recipients of vCJD implicated batches of blood products as being at risk for public health purposes of onward prion transmission. The reputational damage caused to the transfusion services by inadvertent HIV, hepatitis and CJD transmission to patient groups such as hemophiliacs by blood and blood products (e.g., [14]) should, we argue give pause for thought, and dictate a need for reassessment of risks and mitigation strategies when considering new therapeutic modalities such as ACT. Compared to epidemics caused by conventional agents, it is worth reiterating that those involving prions appear to occur in slow motion, with the causes and full recognition of consequences sometimes separated by decades. It, therefore, seems prudent to consider the possibility of prion transmission by novel therapeutic modalities, including ACT, in advance, especially when the therapy is targeted to the brain and eye.

In experimental rodent prion disease models, prions delivered to brain spread to the eye, and those administered intraocularly spread along the retinotectal pathway to the brain ([25], reviewed by Ref. [36]). The involvement of the retina in forms of CJD that originate in the brain [37] and the few documented instances of corneal transplantation-associated iatrogenic CJD [84] argue that similar processes pertain in human prion diseases. It is also now widely accepted that Alzheimer’s disease pathology, notably Aβ deposition, occurs in the retina in Alzheimer’s disease and animal models thereof (reviewed by Ref. [35]) and that this affords opportunities for non-invasive diagnostic and monitoring for Alzheimer’s disease in this accessible part of the central nervous system [45]. Further, formal similarities between Aβ seeding and prion propagation are becoming increasingly accepted (see below) and these similarities include a primary role in disease pathogenesis and disease spread via neuroanatomical pathways (for example [69]). Thus, a formal possibility exists that ongoing efforts to treat non-life-threatening conditions such as blindness arising from retinal diseases by ACT risk facilitation of inadvertent transmission of fatal neurodegenerative conditions such as CJD or other neuroproteinopathies.

## Risk of prion-disease transmission by ACT

In principle, prion or prion-like contamination of ACT could arise by mechanisms mirroring aspects of the known etiologies of CJD itself: acquired, sporadic or genetic. First, it is possible that the contamination could derive from the donation itself. This could be in the form of misfolded protein that remains or propagates along with the cells themselves and is subsequently transmitted to recipients of that particular ACT product. Alternatively, the donated material may harbour an unrecognized mutation that predisposes the encoded gene product to misfold, propagate or be maintained, thus contaminating the ACT product. Second, the cells derived and expanded from the donation may at some point become exposed to exogenous human or animal culture additives that themselves contain prion infectivity, and which replicates or is maintained in the cultured cells and transmitted to the recipients of that particular ACT product. Third, a spontaneous event may occur during the derivation and expansion of the cells, either at the genomic level predisposing the gene product to misfolding or at the epigenetic level by spontaneous protein misfolding that results in a contaminated ACT product. These spontaneous events would be ex vivo analogues of the events that are thought to cause genetic and sporadic CJD, respectively. Last, as with all medical procedures, infectivity could potentially be introduced via contaminated instruments during the course of cell transplantation, a possibility that is analogous to the limited number of iatrogenic CJD cases associated with contaminated surgical instruments. In each of the scenarios described above the subsequent transmission to ACT recipients would be classified as iatrogenic, but the origins (and nature of the infectivity) would have either a sporadic, genetic or acquired etiology. Each of these formal possibilities merits further comment here and we suggest further research in order to quantify and minimize the risk of inadvertent transmission of neuroproteinopathies by ACT.

Recently, the public perception of the risks associated with CJD has been largely driven by the outbreak of BSE [40] and resultant vCJD, which occurred in the UK, and to a lesser extent other European countries [61]. In vCJD, high levels of prion infectivity are more widely distributed throughout the body than in gCJD and sCJD, where it is largely confined to the CNS, and this has been taken to indicate a greater risk of iatrogenic transmission from vCJD as compared to gCJD or sCJD. The absence of evidence of vertical transmission from pregnant mothers with clinical vCJD [60] suggests a degree of protection of the embryo and by inference of derivative hESC. Donor exclusion can reasonably be expected to prevent hESC or other donor-derived cell lines from being established from individuals with or with known family histories of dementia, but it is worth noting that a proportion of cases of gCJD lack a family history and at least some of these are thought to result from de novo mutation of *PRNP*. Despite the more restricted tissue distribution of infectivity in sCJD and gCJD, recent reports indicate that fibroblast, fibroblast-derived hiPSC lines and neuronal derivatives taken from patients with sCJD and gCJD show signs of prion infection [64, 90]. These findings could be interpreted as challenging the perceived idea that prion risks for ACT could be obviated entirely by avoiding cells of UK (and European) origin and concentrating on hiPSC instead of hESC. Irrespective of geographical origin, CJD is exceedingly rare (1–2 cases per million of population per annum) and it would seem statistically unlikely, even in the UK where BSE infection has been estimated to be as high as 1:2000 of the general population [29], that an ACT would be based on cells derived from an individual with clinical or asymptomatic vCJD.

The possibility that exogenous prion contamination could occur during human cell processing (i.e., derivation, expansion, differentiation, purification, packaging for delivery outside of the body) also warrants consideration. A priori, culture additives of human or bovine origin or other reagents exposed to reagents of human or bovine origin in their preparation would seem to present the highest potential risks. All animal reservoirs of prion infectivity should be considered including those whose risk of transmission to humans remains relatively unexplored such as dromedary camels and deer [3, 87]. As regards the latter, CWD in deer originating in North America constitutes a rapidly spreading epidemic with the potential to spread to humans arising from the consumption of wild game. In recent years CWD has arrived in continental Europe [6].

**Fig. 2:**
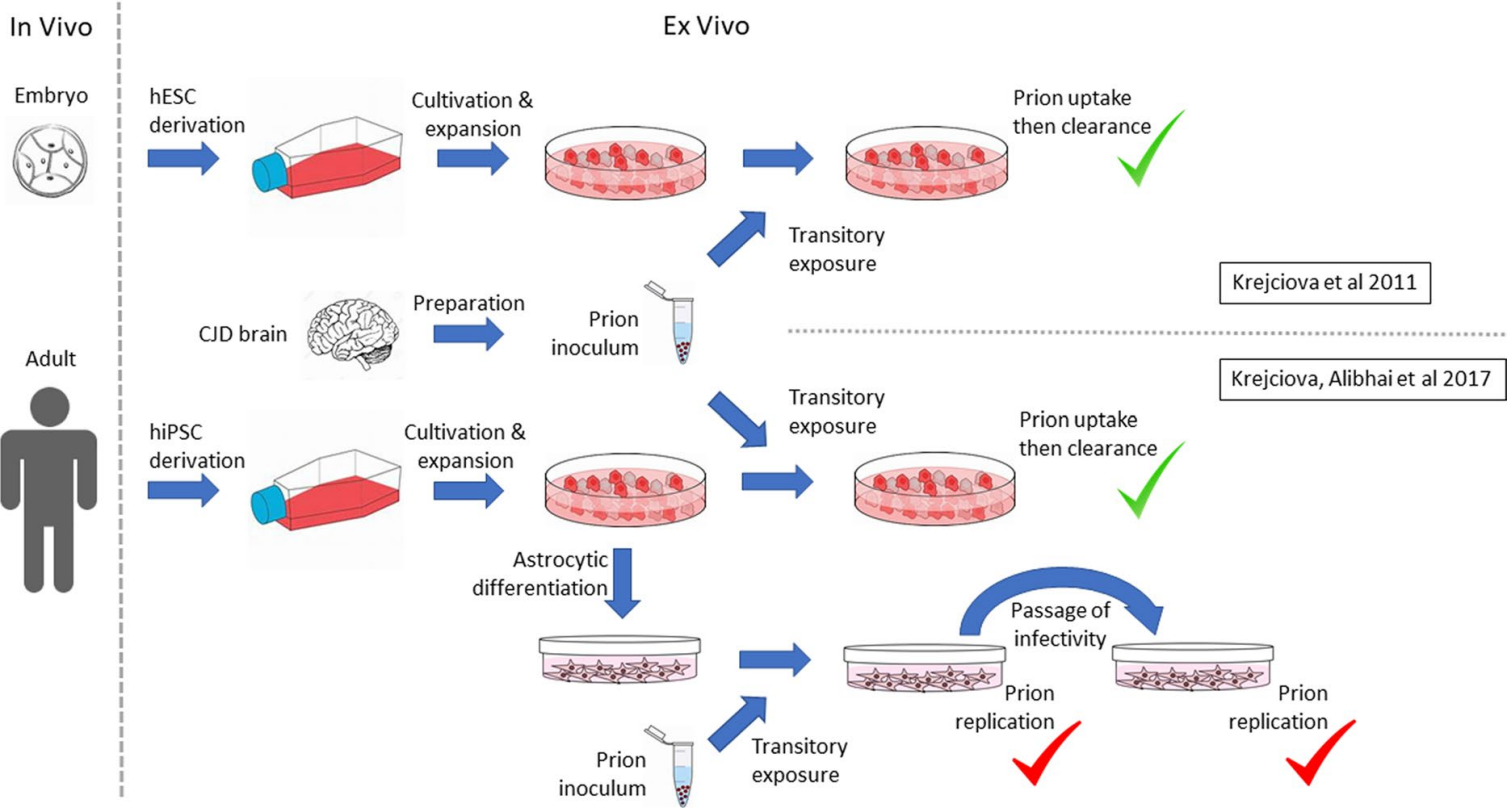
Prion infection of human embryonic and induced pluripotent stem cells and their astrocyte derivatives. Schematic representation of design and outcome of author-led studies reflecting limited published knowledge on the susceptibility to and replication of infectious prions in pluripotent and derivative cells, namely [47, 48]

We have previously shown that hESC take up and then rapidly clear abnormal prion protein in BSE, vCJD and sCJD brain homogenates experimentally inoculated into the culture medium (Fig. 2, [48]). Clearance is reassuring from a safety point of view, nevertheless we noted three major caveats at the time of this work: first, these were proliferating hESC, which might dilute out slowly replication prions. Second, the methods used to detect prions were of relatively low analytical sensitivity. Third, these were stem cells and not the derivative therapeutic products to be transplanted in emerging ACT. More recently, we have shown that astrocytes differentiated in vitro by an established method [77] from human-induced pluripotent stem cells (hiPSC) from normal donors are susceptible to infection with human prions from sCJD and vCJD brain in a genotype-dependent manner and that the infection is not transient and can be passed from one astrocyte culture to another (Fig. 2, [47]). This raises the possibility that overt prion replication may be differentiation dependent and that low-level prion contamination may only become evident when appropriate cell differentiation occurs on therapeutic application.

CJD associated with de novo *PRNP* mutations does occur in vivo in human populations, albeit at a very low incidence. However, at this time there is little evidence on which to base an evaluation of risk of spontaneous development of prion or prion-like infectivity ex vivo in the course of ACT cell processing, except that it is reasonable to assume that the likelihood of acquiring a pathogenic mutation in *PRNP* will increase as a factor of time in culture and the number of rounds of cell division prior to therapeutic use. During cell culture, perturbation of endoplasmic reticulum (ER) homeostasis can facilitate prion replication [39] and chronic or acute ER stress is a de facto risk of extensive cell processing. Thus, ACTs founded on extensively processed cell resources are more likely to experience sub-optimal culture conditions or handling inducing ER stress. They would thus theoretically carry a greater risk of spontaneous protein misfolding and the potential to replicate otherwise undetectable prion contaminants than minimally processed cells.

There is no obvious reason to presume that the physical introduction of ACT products to patients bears greater risks of infection through the use of contaminated surgical instruments than other comparable surgical interventions. Nevertheless, it is important to recognize that neurosurgery, ocular surgery in the form of corneal transplantation, transfusion and intramuscular inoculation are all recognized routes of iCJD transmission (Table 2) and careful consideration should be given to the challenging physical properties of prions and prion-like infectivity when selecting appropriate decontamination regimes [5, 22, 81].

## Prion-like mechanisms and other protein misfolding disorders

The hypothesis that CJD and other transmissible spongiform encephalopathies result from self-propagating conformational variants of the prion protein yielding a transmissible amyloidosis (the prion hypothesis) has in the past decade gone through a dramatic expansion. This owes to observations of prion-like behaviors of other proteins, such as beta amyloid (Aβ), tau, α-synuclein and TDP-43 that are proposed to underlie neurodegenerative diseases as diverse as Alzheimer’s disease, Parkinson’s disease, multiple systems atrophy, frontotemporal dementia and motor neuron disease, respectively. The case for reframing these diverse neurodegenerative disorders explicitly as prion diseases has been made [67, 68]. The original observations on which this assertion is based derive from cell culture and transgenic animal studies showing that aspects of the molecular pathology that characterize each disease and its spread between cells, around the brain and even between individual animals share fundamental similarities with those already defined in prion diseases. Intracerebral inoculation of Aβ containing brain extracts into amyloid precursor protein (APP) transgenic mice was first shown to be sufficient to result in an Aβ amyloidosis in the recipient animals [55]. The analogy with experimental prion disease transmission was further strengthened by the observations that cerebral Aβ amyloidosis could result from peripheral (in addition to central) inoculation and that phenotypic differences occurred dependent on the source of the inoculum and genotype of the recipient, a situation reminiscent of prion strains [55, 20]. Intracerebral inoculation of brain extracts from mice expressing disease-associated tau mutations appears sufficient to transmit a spreading tauopathy in mice expressing wild-type tau [12]. Similarly, intracerebral inoculation of brain extracts from patients with multiple systems atrophy (MSA) in mice hemizygous for a mutant α-synuclein gene appears sufficient to produce a progressive neurological disease sharing molecular and cellular features with MSA [88], a finding that extended the original observation that intracerebral inoculation of synthetic α-synuclein fibrils can precipitate Parkinson’s disease-like pathology and neurodegeneration in non-transgenic mice [52]. Structural variants of abnormal α-synuclein and tau have also been described that may account for different disease phenotypes and hence might be interpreted as the basis of prion-like strain effects [7, 24]. These observations have led to a working hypothesis that many of the most common neurodegenerative conditions are in fact neuroproteinopathies that share a common molecular mechanism in which different proteins (Aβ, tau, α-synuclein and perhaps others) function as “propagons”, seeding the conversion of their normal cellular counterparts to the abnormal disease-associated isoform and thus spreading the pathological process between cells and around the brain. Moreover, the finding that propagons of a given protein can exist in multiple different “conformers” or “isotypes” that “seed” or “template” with fidelity may be taken to be indicative of strain-like properties. The experimental data in this area are complex and subject to differing interpretations, but it has been critically and authoritatively reviewed recently at length in a cluster of articles in this journal [19, 50, 85].

**Fig. 3:**
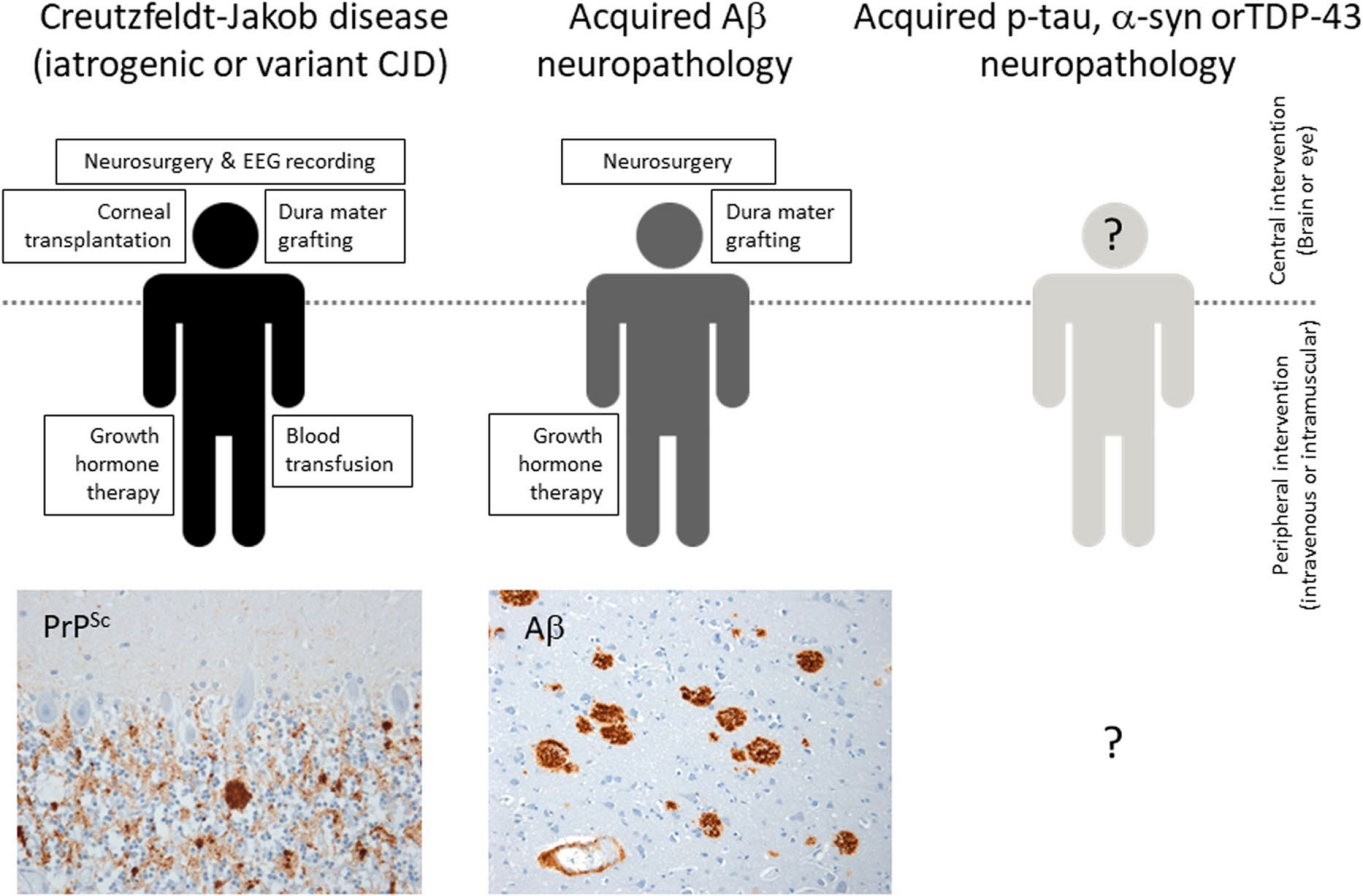
Currently known or suspected routes of human-to-human neurodegenerative disease or associated protein pathology transmission occurring as a consequence of medical treatment. Iatrogenic Creutzfeldt–Jakob disease and its associated PrP^Sc^ deposition has been shown to occur following interventions in the brain and eye and via peripheral exposure in recipients of human growth hormone. Variant Creutzfeldt–Jakob disease has also been transmitted through blood transfusion. Immunostaining for disease associated prion protein (PrP^Sc^, brown) in the cerebellum of a recipient of human growth hormone is shown. Recently, amyloid beta (Aβ) neuropathology has been shown to occur in several distinct iatrogenic Creutzfeldt–Jakob disease cohorts associated with both central and peripheral exposure to prion infectivity. The presence of Aβ neuropathology in human growth hormone recipients who did not develop Creutzfeldt–Jakob disease indicates that Aβ neuropathology is independently transmissible. Aβ deposition (brown) in the cerebral cortex and cerebral blood vessels of one such patient is shown. Acquired phospho-tau (p-tau), α-synuclein (α-syn) or TDP-43 neuropathology have yet to be demonstrated

The possibility of actual disease transmission (acquired Alzheimer’s disease for example) between individuals is more controversial and currently lacking an epidemiological evidence base (see for example [18]); nevertheless, it cannot be excluded at this point in time. Strong evidence that Aβ protein pathology (as distinct from Alzheimer’s disease) is transmissible between people has recently appeared and again this has come from CJD research. The idea that pituitary-derived human growth hormone recipients might have been exposed to pathological proteins from Alzheimer’s or Parkinson’s disease brains (in addition to prion protein from CJD brain) was raised by Irwin et al. [41], demonstrated by Jaunmuktane et al. [42] and confirmed and extended by us [70] and others [9, 17]. Similarly, an apparently acquired cerebral Aβ angiopathy has been shown to be present in iatrogenic CJD cases linked to dura mater grafting [9, 26, 34, 46] and in a small number of adult individuals without CJD, but who had neurosurgery in childhood [43]. The recent finding of a fatal Aβ amyloid angiopathy in a patient four decades after dura grafting and with no evidence of CJD is of particular significance [38]. The current state of knowledge regarding known transmissions of prion and prion-like pathologies in humans is summarized in schematic form with examples in Fig. 3 and documented in detail in Table 2. Taken together and in combination with the precautionary principle, the implications of these data include the proposition that ACT risk assessment should consider the possibility of transmission of more common proteinopathies, in addition to CJD.

## Prion testing of ACT products

The development of methods to screen for prions in donated blood has been fraught with difficulties [14]. This is in part because of technical challenges: the absence of nucleic acid component to the agent, the shared antigenicity between host and agent prion protein and the very low limit of detection required for detection in blood from asymptomatic individuals. Difficulties have also arisen because the most relevant clinical samples for test validation are not readily available and because any candidate blood screening assay must be both high throughput and rapid to satisfy transfusion service need. Not all of the above considerations apply to prion testing of ACT products, but we appear to be approaching a tipping point in analytical sensitivity in prion assays with reports that techniques such as protein misfolding cyclic amplification (PMCA) or real-time quaking-induced conversion (RT-QuIC) can detect prions in blood, CSF or urine from vCJD or sCJD patients [4, 15, 49, 54, 58]. Moreover, such techniques may be amenable to modifications that allow similarly sensitive and specific detection of other neurodegenerative disease-associated misfolded protein such a Aβ and α-synuclein [21, 73, 78]. Rather than waiting for a validated test to appear that is optimized for blood transfusion service need, we suggest that now is the right time to apply existing technologies to the specific needs of developing ACT products.

## Risk mitigation

Whether or not the prion or prion-like risk is readily amenable to precise assessment by emerging methods, we suggest that even a single ACT-mediated transmission of CJD or prion-like neurodegenerative disease could be catastrophic for confidence in the emerging field of ACTs generally. It is tempting to suggest that CRISPR/Cas9 mediated deletion of *PRNP* from a prospective ACT product at the time of derivation would offer the best protection from prion disease transmission, since cellular prion protein expression is a precondition for prion propagation and neurotoxicity (reviewed by Ref. [2]). This would also serve to prevent the in situ spread of protein pathology from affected host cells to unaffected therapeutic cells as seen in grafted Parkinson’s disease patient brains [51] and would, therefore, ensure continued ACT efficacy. Unfortunately, there is increasing evidence that the prion protein itself performs roles in the maintenance and differentiation of stem cells (reviewed by Refs. [33, 57]), so its deletion may have unpredictable and undesirable effects with respect to ACTs. Instead, we suggest that there are a series of reasonable and practicable precautionary steps to which the community of ACT developers and regulators supported by public and private resources should commence commitment to the following:

1. Careful re-evaluation of ACT cell donor medical and family history selection criteria and emerging scientific understanding to consider risk of transmitting prion and prion-like proteinopathies, including, but not limited to, risk factors associated with Alzheimer’s disease, Parkinson’s disease, multiple systems atrophy and frontotemporal dementias, in addition to Creutzfeldt–Jakob disease.
2. Locus-focused gene sequencing of all candidate ACT cell resources and derivative therapeutic products for mutations associated with known pathogenic prion isoforms and prion-like proteinopathies.
3. Continued optimization of derivation and cell expansion conditions that avoid as far as possible biologicals of human and animal origin.
4. Commitment to develop and apply high sensitivity detection methods such as variations of PMCA and RT-QuIC for prion and prion-like proteinopathies for purposes of Quality Control and Release of manufactured ACT products.
5. Integral to the previous, development of reference standards to qualify detection of prion and prion-like proteinopathies in ACT products and their dissemination in support of international harmonization of standards for the testing ACTs.
6. Basic research on the extent to which cell processing steps critical for ACT manufacture impacts on spontaneous mutations or misfolding promoting prion or prion-like replication and infectability.

## Conclusion

In summation, the available evidence substantiates the perspective that the risk of transmitting prion or prion-like proteinopathies with modern day ACT for neurological or other diseases is still likely to be remote. However, historical precedent and developments in the field in recent years does justify reassessment of precautionary measures, as outlined. Such effort would be consistent with the long-standing commitment of ACT developers, regulators and public and private investors to assure the future safety and efficacy of emerging therapeutic interventions for debilitating diseases yet to be effectively treated or cured by other approaches.

## Search strategy and selection criteria

References used herein were identified by searches of PubMed on the 15th of June 2018 or previously known to the authors. The search terms “Cell Therapy”, “Prion”, “Prion-like”, “Prionopathy” and “Proteinopathy” were used. Cell therapies currently under clinical evaluation (Phase I–IV) were identified on the same date by searching the US international registry of clinical trials [13]; The final reference list was generated on the basis of relevance to the topics covered in this Review.

## Electronic supplementary material

Below is the link to the electronic supplementary material.

Supplementary material 1 (DOCX 19 kb)

## Abbreviations

Aβ: Amyloid beta
ACT: Advanced cell therapy
APP: Amyloid precursor protein
BSE: Bovine spongiform encephalopathy
CJD: Creutzfeldt–Jakob disease
CRISPR/Cas9: Clustered regularly interspaced short palindromic repeats/CRISPR associated protein 9
CNS: Central nervous system
CSF: Cerebrospinal fluid
CWD: Chronic wasting disease
ER: Endoplasmic reticulum
hESC: Human embryonic stem cells
hGH-iCJD: Human growth hormone-associated iatrogenic Creutzfeldt–Jakob disease
hMSC: Human mesenchymal stem cells
iCJD: Iatrogenic Creutzfeldt–Jakob disease
hiPSC: Human induced pluripotent stem cells
MSA: Multiple systems atrophy
PMCA: Protein misfolding cyclic amplification
PrP^Sc^: Disease-associated isoform of the prion protein
*PRNP*: Human prion protein gene
RT-QuIC: Real-time quaking induced conversion
sCJD: Sporadic Creutzfeldt–Jakob disease
TDP-43: TAR DNA binding protein 43
USA: United States of America
vCJD: Variant Creutzfeldt–Jakob disease
