# Supplementary material for: Renewed assessment of the risk of emergent advanced cell therapies to transmit neuroproteinopathies
Source: Acta Neuropathol. 2018 Nov 27;137(3):363–77. doi: 10.1007/s00401-018-1941-9 (PMC6514076; doi:10.1007/s00401-018-1941-9)
Supplement: Supplementary file 1 — Supplementary material 1 (DOCX 19 kb) [file 401_2018_1941_MOESM1_ESM.docx]

**Renewed assessment of the risk of emergent advanced cell therapies to transmit neuroproteinopathies**

Paul A. De Sousa ^1,‡^, Diane Ritchie^1,2^, Alison Green ^1,2^, Siddharthan Chandran ^1^, Richard Knight ^1,2^, Mark W. Head ^1,2,^

^1^Centre for Clinical Brain Sciences, University of Edinburgh, Chancellors Building, 49 Little France Crescent, Edinburgh, UK, EH16 4SB.

^2^ National CJD Research & Surveillance Unit, Western General Hospital, Crewe Road, Edinburgh, EH4 2XU.

**List of Supplementary Tables Page**

**Table S1** Tabulation of [www.clinicaltrials.gov](http://www.clinicaltrials.gov) search terms and links………………………………………………..**2**

**Table S1. Search date, terms and links to outcomes of** [**www.clinicaltrials.gov**](http://www.clinicaltrials.gov) **searches.**

| Search | Date | Terms | Link |
| --- | --- | --- | --- |
| 1 | 15 June 2018 | “Allogeneic Mesenchymal”; Recruiting; Completed; Phase early 1, 1, 2 | <https://www.clinicaltrials.gov/ct2/results?cond=&term=&type=&rslt=&recrs=a&recrs=e&age_v=&gndr=&intr=allogeneic+mesenchymal&titles=&outc=&spons=&lead=&id=&cntry=&state=&city=&dist=&locn=&phase=4&phase=0&phase=1&strd_s=&strd_e=&prcd_s=&prcd_e=&sfpd_s=&sfpd_e=&lupd_s=&lupd_e>= |
| 2 | 15 June 2018 | “Allogeneic Mesenchymal”; Recruiting; Completed; Phase 3, 4 | <https://www.clinicaltrials.gov/ct2/results?cond=&term=&type=&rslt=&recrs=a&recrs=e&age_v=&gndr=&intr=allogeneic+mesenchymal&titles=&outc=&spons=&lead=&id=&cntry=&state=&city=&dist=&locn=&phase=2&phase=3&strd_s=&strd_e=&prcd_s=&prcd_e=&sfpd_s=&sfpd_e=&lupd_s=&lupd_e>= |
| 3 | 15 June 2018 | “Allogeneic Cell Line, Recruiting; Completed; Phase early 1, 1, 2 | <https://www.clinicaltrials.gov/ct2/results?cond=&term=&type=&rslt=&recrs=a&recrs=e&age_v=&gndr=&intr=allogeneic+cell+line&titles=&outc=&spons=&lead=&id=&cntry=&state=&city=&dist=&locn=&phase=4&phase=0&phase=1&strd_s=&strd_e=&prcd_s=&prcd_e=&sfpd_s=&sfpd_e=&lupd_s=&lupd_e>= |
| 4 | 15 June 2018 | “Allogeneic Cell Line”; Recruiting; Completed; Phase 3, 4 | <https://www.clinicaltrials.gov/ct2/results?cond=&term=&type=&rslt=&recrs=a&recrs=e&age_v=&gndr=&intr=allogeneic+cell+line&titles=&outc=&spons=&lead=&id=&cntry=&state=&city=&dist=&locn=&phase=2&phase=3&strd_s=&strd_e=&prcd_s=&prcd_e=&sfpd_s=&sfpd_e=&lupd_s=&lupd_e>= |
| 5 | 15 June 2018 | “Embryo Stem Cell”; No specification of recruitment status; Phase early 1, 1, 2, 3, 4 | <https://www.clinicaltrials.gov/ct2/results?term=embryo+stem+cell&phase=01234> |
| 6 | 15 June 2018 | “Induced pluripotent stem cells” with no specification of recruitment status or phase status | <https://www.clinicaltrials.gov/ct2/results?cond=&term=induced+pluripotent+stem+cells&type=&rslt=&age_v=&gndr=&hlth=Y&intr=&titles=&outc=&spons=&lead=&id=&cntry=&state=&city=&dist=&locn=&strd_s=&strd_e=&prcd_s=&prcd_e=&sfpd_s=&sfpd_e=&lupd_s=&lupd_e>= |
